# Efficacy of a 2-week therapy with levofloxacin concomitant versus a levofloxacin sequential regimen for *Helicobacter pylori* infection in the Syrian population: a study protocol for randomized controlled trial

**DOI:** 10.1186/s13063-024-07906-3

**Published:** 2024-01-15

**Authors:** Marouf Alhalabi, Rasha Almokdad

**Affiliations:** Gastroenterology Department, Damascus Hospital, Almujtahed Street, Damascus, Syria

**Keywords:** *Helicobacter Pylori*, Levofloxacin, Esomeprazole, Amoxicillin, Metronidazole, Tinidazole, Proton bump inhibitor, Concomitant, Sequential, Syria

## Abstract

**Background:**

Treating *Helicobacter pylori* is becoming increasingly difficult with the development of bacterial resistance to many established treatment regimens. As a result, researchers are constantly looking for novel and effective treatments. This trial aims to establish the efficacy of levofloxacin-based sequential treatment regimen and concomitant levofloxacin-based regimen as empirical first-line therapy in the Syrian population.

**Method:**

This is an open-label, prospective, single-center, parallel, active-controlled, superiority, randomized clinical trial. The recruitment will target *Helicobacter pylori*-positive males and females between the ages of 18 and 65 to evaluate the efficacy of empirical first-line therapy in the Syrian population. We are planning to recruit up to 300 patients which is twice the required sample size. One hundred fifty individuals will be randomly assigned to undergo either a sequential levofloxacin-based treatment regimen or a concomitant levofloxacin-based regimen. High-dose dual therapy (proton-pump inhibitor and amoxicillin) will be the rescue therapy in the event of first-line failure. The first-line eradication rate in both groups is the primary outcome, and one of the secondary outcomes is the overall eradication rate of high-dose dual therapy in the event of first-line treatment protocol failure. Intention-to-treat analysis and per-protocol analysis will be used to evaluate the eradication rates of *Helicobacter pylori* for first-line treatment protocols.

**Discussion:**

For the first time in the Syrian population, this randomized controlled trial will provide objective and accurate evidence about the efficacy of a sequential levofloxacin-based treatment regimen.

**Trial registration:**

ClinicalTrials.gov NCT06065267. Registered on October 3, 2023. Prospective registered. Enrollment of the first participant has not started yet.

**Supplementary Information:**

The online version contains supplementary material available at 10.1186/s13063-024-07906-3.

## Administrative information

Note: the numbers in curly brackets in this protocol refer to SPIRIT checklist item numbers [1]. The order of the items has been modified to group similar items [2] (see http://www.equator-network.org/reporting-guidelines/spirit-2013-statement-defining-standard-protocol-items-for-clinical-trials/).

**Table:** 

Title {1}	Efficacy of a two-week therapy with levofloxacin concomitant versus a levofloxacin sequential regimen for Helicobacter pylori infection in the Syrian population: a study protocol for randomized controlled trial
Trial registration {2a and 2b}	ClinicalTrials.gov: Identifier:(NCT06065267 direct link https://clinicaltrials.gov/study/NCT06065267). Efficacy of a two-week therapy with levofloxacin concomitant versus a levofloxacin sequential regimen for Helicobacter pylori infection in the Syrian population: a study protocol for randomized controlled trial
Protocol version {3}	Protocol version 3.0, December 24,2023
Funding {4}	Scientific research fund of Damascus Hospital, No. GD-23091
Author details {5a}	All authors are affiliated with Damascus Hospital
Name and contact information for the trial sponsor {5b}	Grantee and trial sponsor: Damascus Hospital Contact name: Dr.Ahmad Abbas, general manager of Damascus hospital Address: No. Syria, Damascus, Almujtahed Street, Damascus Hospital Telephone: + 963992490396 E-mail: ahmadJabbas@outlook.com
Role of sponsor {5c}	The grantee and sponsor (Damascus Hospital) will not be a part of the trial procedures (including trial design, gathering data, data management, data analysis and interpretation, report writing, and publication decision). The sponsor has no authority over any of the mentioned activities

## Introduction

### Background and rationale {6a}

Unfortunately, there is no information on the prevalence of *Helicobacter pylori* (HP) infection among Syrians [3]. A systematic review showed that the prevalence of HP infection ranges between 22 and 87.6% in Middle Eastern countries; regrettably, it did not include any data from Syria [4]. Syrian refugees may have an HP infection prevalence similar to that of their native country. There are two reports of the prevalence of HP infection among Syrian refugees. The first reported that only 8 individuals (66.7%) from the Middle East region were infected with HP when they presented to a family care clinic in the USA. Unfortunately, this prevalence includes patients from across the Middle East and may not adequately reflect the prevalence of infection among Syrians [5]. The second report from Germany revealed that the prevalence of HP infection among Syrian refugees was about 34%, which may be closest to reality [6]. The prevalence of HP infection appears to be higher in developing nations than in industrialized nations, with the majority of infections happening during childhood. Poor sanitation standards, low-income levels, and overcrowded living conditions appear to be associated with a higher prevalence of HP infection [7]. The recent humanitarian crisis has had a terrible impact on Syrian lives, resulting in millions of refugees and displaced individuals, massive infrastructure destruction, and the greatest economic catastrophe Syria has ever faced. It had an enormous impact on the health sector, with up to 50% of health facilities destroyed and up to 70% of healthcare providers fleeing Syria [8, 9]. Peptic ulcer disease and consequent bleeding [10–12], gastric adenocarcinoma [13, 14], dyspepsia [15, 16], mucosa-associated lymphoid tissue (MALT) lymphoma [17, 18], unexplained iron deficiency anemia [19], and idiopathic thrombocytopenic purpura [20, 21] are all linked to HP infection, which requires antimicrobial treatment [22, 23]. In real-world applications, only a few antibiotics are efficient at eradicating HP infection. Treatment regimens include a combination of two or three antibiotics, and a proton pump inhibitor (PPI), with or without a bismuth component that gives extra antibiotic properties [22, 23]. However, the increasing antibiotic resistance of HP has become a major global problem [24–35]. In Syria, the eradication rate of traditional triple therapy with clarithromycin or levofloxacin was less than 30% [36], whereas the eradication rate with the levofloxacin concomitant regimen and the doxycycline-bismuth-based quadruple regiment was 82.05% and 78.9%, respectively [37]. As a result, there is a need to look for more effective therapeutic regimens as well as the best therapeutic regimens for follow-up when the first line of treatment fails.

## Objectives {7}

The main objective of this study is to compare the eradication rate of HP infection using levofloxacin-based sequential therapy against levofloxacin-based concomitant therapy using an intention-to-treat analysis (ITT) and a per-protocol analysis (PPA).

## Trial design {8}

**Fig. 1:**
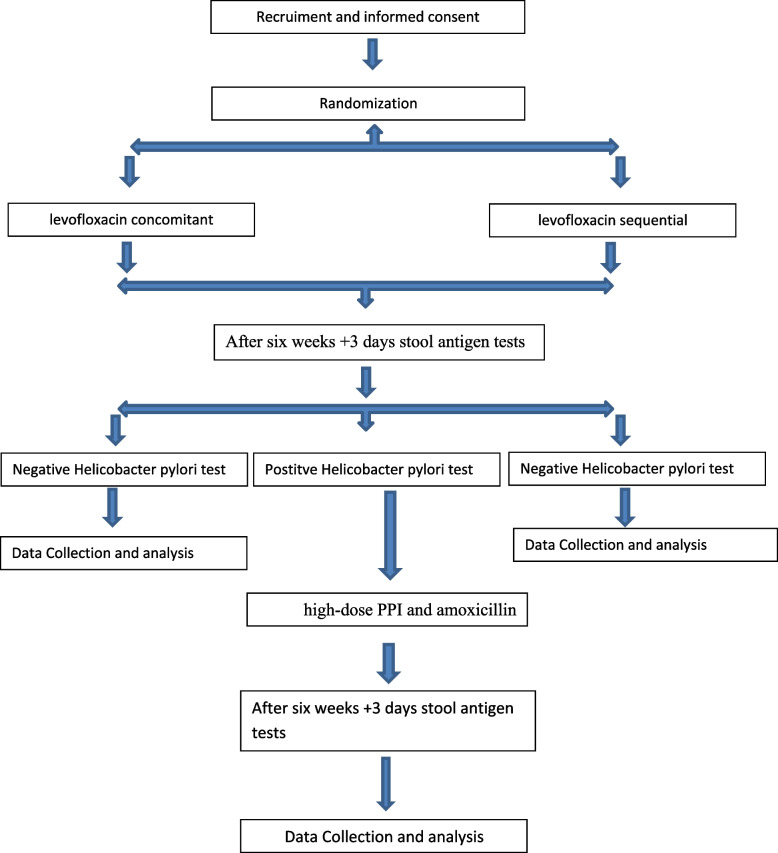
Trial flowchart

This is a single-center, prospective, superiority randomized, open-label, active-controlled clinical trial with a 1:1 allocation ratio. Figure 1 shows a trial flow chart.

## Methods: participants, interventions, and outcomes

### Study setting {9}

The research will be carried out in Damascus Hospital’s outpatient clinic. Damascus Hospital is the primary medical facility affiliated with the Ministry of Health. Damascus, Syria’s capital, treats patients from throughout the country.

### Eligibility criteria {10}

#### Inclusion criteria

(1) Men and women between the ages of 18 and 65 years; (2) naive to HP infection treatment; (3) HP-positive, as determined by histological examination.

#### Exclusion criteria

(1) Allergic to any medicine supplied; (2) pregnant or lactating; (3) suffering from serious systemic disorders, such as severe cardiopulmonary or hepatic dysfunction; (4) having had a previous gastrectomy or a history of stomach cancer; (5) chronic renal failure; (6) unwilling to participate in the trial; (7) persistent use of non-steroidal anti-inflammatory drugs (NSAIDs), antibiotics, proton-pump inhibitors (PPIs), H_2_ receptor antagonists, aspirin, herbal remedies, and probiotics during the trial procedure. Keeping to take any dose of any previous medication is compatible with the concept of persistence, and any participant who also starts using or uses any of these treatments during the trial or follow-up will be excluded [38].

### Who will take informed consent? {26a}

Before beginning any trial procedures, investigators must get written informed consent from patients. The investigators will explain and discuss the experiment with potential volunteers to ensure that they understand what is being studied and that their involvement is entirely voluntary. Patients will be informed that they can drop out of the trial at any time. It was explicitly specified that only clinical information would be discussed in the research, with no private data mentioned in any part of the trial report. It was made clear to all patients that dropping out of the trial would not affect the quality of follow-up or treatment.

### Additional consent provisions for collection and use of participant data and biological specimens {26b}

A written request form must accompany every specimen, and the identification information on the specimen and requisition must be identical. The requisition form must contain all the following information: (1) patient’s legal name, (2) unique identification number, (3) age, (4) source of the specimen, (5) complete provider details, (6) underlying medical condition, and (7) pathology investigations requested. All specimen containers must be leak-proof, placed in a secondary leak-proof container for transport to the laboratory, and transported to the laboratory as quickly as possible. Tissue specimens must be suspended or totally immersed in ten times their volume of 10% neutral-buffered formalin to maintain the integrity of the specimen. The initial informed consent form contains data collection and request information.

### Interventions

#### Explanation for the choice of comparators {6b}

For two weeks, patients on the concomitant levofloxacin regimen will receive levofloxacin 500 mg once daily, amoxicillin 1000 mg, tinidazole 500 mg, and esomeprazole 20 mg twice daily. When applied as a first-line treatment, the previous regimen had the highest eradication rate for Syrian patients naive to HP treatment [36, 37]. A rescue regimen of high-dose dual therapy consisting of esomeprazole (40 mg twice daily) and amoxicillin (1000 mg three times daily) for 2 weeks will be used after first-line treatment fails [22, 39].

#### Intervention description {11a}

The sequential levofloxacin regimen consists of esomeprazole 20 mg and amoxicillin 1000 mg taken twice daily for 1 week, followed by metronidazole 500 mg, esomeprazole 20 mg twice daily, and levofloxacin 500 mg once daily for 1 week [22, 40]. Microscopic examination of the stomach biopsies and hematoxylin and eosin, followed by Giemsa stains [41], will be used to confirm HP infection, which has a sensitivity and a specificity of 95% and 99%, respectively [41]. Gastric biopsies will be collected via gastroduodenoscopy and forwarded to the pathology laboratory of the same referral hospital. According to the Sydney system [42], endoscopists obtained five stomach biopsies: two from the body, two from the antrum, and one from the incisura. If the first-line treatment fails, a high-dose dual therapy of PPI and amoxicillin will be provided as a rescue therapy [22].

#### Criteria for discontinuing or modifying allocated interventions {11b}

The safety of HP eradication therapy is well known, with the most common adverse events being taste disturbance, diarrhea, nausea, and abdominal pain. The vast majority of adverse events are minor and transient [43]. According to estimates, just 1.3% of patients terminate treatment due to adverse events [44]. Six weeks after ending treatment, all patients will visit the central laboratory of our hospital and undergo stool antigen tests using the enzyme immunoassay method (EIA) [45]. In our research, when individuals report adverse events, the investigators will examine them. The experiment’s drugs may be withdrawn for any of the following reasons: (1) significant adverse events that are considered unsuitable for continuing treatment, such as events that are life-threatening, necessitate inpatient hospitalization, result in persistent or significant disability or incapacity, or may necessitate medical or surgical intervention to prevent one of the outcomes listed above; (2) inability to comply with the trial procedures. Individuals may withdraw from the trial at any time for any reason. The reasons for withdrawal will be noted if individuals indicate them.

#### Strategies to improve adherence to interventions {11c}

Face-to-face treatment and leaflet instruction about the trial will be provided at the initial appointment such as how to take the medication, the potential treatment interactions, side effects, and contraindications; any inquiries will be welcomed either in person by visiting investigators or over the phone. Investigators will discuss the importance of participants completing treatment regimens. Patients are also encouraged to alert investigators if they experience issues linked to trial therapies. Furthermore, participants will be given instructions on how to take trial medications (dosage, timing, and storage), as well as what to do if a dose is missed. Participants will also get a compliance reminder phone call twice a week after treatment begins.

#### Relevant concomitant care permitted or prohibited during the trial {11d}

All other antibiotics, proton-pump inhibitors (PPIs), H_2_ receptor antagonists, non-steroidal anti-inflammatory drugs (NSAIDs), aspirin, herbal remedies, and probiotics will be restricted during the trial procedure.

#### Provisions for post-trial care {30}

As this trial is a low-risk intervention, no particular post-trial care is required. The trial’s risks are covered by insurance at the trial site. It may involve additional health care coverage, reimbursement, or damages.

### Outcomes {12}

The trial’s primary outcome is the percentage of patients who have successfully eradicated HP infection. This will be established based on the findings of stool antigen testing using the enzyme immunoassay method (EIA) [45], 6 weeks after the completion of the first-line treatment phase of the levofloxacin-based concomitant or levofloxacin-based sequential treatment regimen. The HP stool antigen test is an accurate approach for confirming HP eradication after the fourth week following treatment, based on a comprehensive evaluation of 25 reports involving 2078 individuals who examined the HP stool antigen test for confirmation of HP eradication. The sensitivity, specificity, positive predictive value (PPV), and negative predictive value (NPV) were as follows: 88.3%, 92%, 75.1%, and 94.8% [46].

The secondary outcomes are:

1. The rate of HP eradication of rescue treatments and total eradication rates among the levofloxacin-based concomitant and levofloxacin-based sequential treatment regimens; (2) the type and frequency of adverse events, as well as the rate of compliance, between the levofloxacin-based concomitant and levofloxacin-based sequential treatment regimen groups.

### Participant timeline {13}

**Fig. 2:**
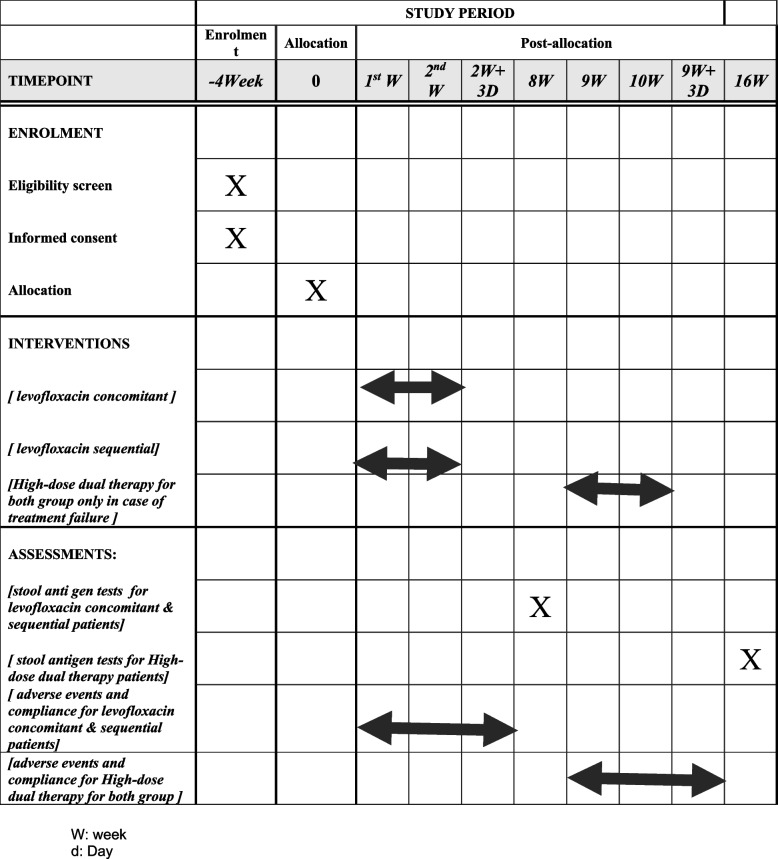
Study schedule of enrollment, intervention, and assessments

Figure 2 summarizes the enrollment, intervention, and assessment timeline.

### Sample size {14}

We will conduct this as a superiority clinical trial, and the calculation of the sample size is based on the primary outcome, which is the eradication rate of HP infection using ITT. According to a meta-analysis, Kale-Pradhan et al. reported the eradication rate of a sequential levofloxacin-based treatment regimen as 87.8% (P2 = 0.877) [40], while the eradication rate of a concomitant levofloxacin-based regimen was based on the results of a randomized clinical trial conducted in Syria and was 82.05% (P1 = 0.8205) [37]. We applied the following statistical hypotheses: (1) 80% power (*β* = 0.2); (2) the 5% level of significance (*α* = 0.05); and (3) the superiority margin is 10% (*δ* =  − 0.10) and (*ε* = 0.20). Given that *Zα* and *Z*_*β*_ are 1.64 and 0.845, respectively; (4) the ratio of case to control is equal to one (*K* = 1). The sample size formula of the two parallel superiority designs resulted in 64 patients as a sample size for each parallel [47, 48]. We also added 15% for possible dropouts, making the final sample size 150 patients. For more information, please check Additional file 1.

### Recruitment {15}

Participants will be recruited through Damascus Hospital’s outpatient clinic. We also have a dedicated phone line to answer any questions about participating in the study. On average, our outpatient clinic sees 10 to 15 newly diagnosed HP-infected patients per month. The investigators will explain the trial to eligible patients and ensure they understand the hazards of participating. Before taking part in the experiment, patients will need to sign an informed consent form.

## Assignment of interventions: allocation

### Sequence generation {16a}

An independent assistant will generate a randomized number table using LibreOffice Calc’s RANDBETWEEN function [49].

### Concealment mechanism {16b}

The independent assistant will keep the randomized number table sealed in an envelope.

### Implementation {16c}

Once a researcher has obtained the patient’s informed consent, they will contact the independent assistant to get the allocated treatment regimen.

## Assignment of interventions: blinding

### Who will be blinded {17a}

It is challenging to blind the participants and the researchers in this open-label trial. However, throughout the whole trial, laboratory medical professionals, data collectors, and data analysts will remain blinded to the therapy allocation. The allocation information will be hidden on a data collection form for adverse events and compliance, and data collectors are not permitted to inquire about participants' regimens.

### Procedure for unblinding if needed {17b}

Not applicable because it is not possible to blind the investigators or participants.

## Data collection and management

### Plans for assessment and collection of outcomes {18a}

During screening, sociodemographic and baseline information (including age, sex, contact information, and past medical history) will be obtained. The success of HP eradication will be assessed by stool antigen assays performed with the enzyme immunoassay method (EIA) 6 weeks following the completion of therapy [45]. Participants will be prohibited from using PPIs, antibiotics, H_2_ receptor antagonists, aspirin, herbal remedies, and probiotics for 8 weeks until the stool antigen testing. The stool antigen tests must be performed no later than 6 weeks + 3 days after the completion of therapy. The data on adverse events and compliance will be obtained face-to-face and documented using the data collection form no later than 3 days following the completion of therapy. The count of pills taken will be used to determine study drug compliance. The data will be obtained from two trained assistants who are instructed to collect the information in a consistent, reproducible manner, and the integrity of the data will be overseen by the principal investigator.

### Plans to promote participant retention and complete follow-up {18b}

Investigators will communicate with participants on a regular basis by phone and WhatsApp twice a week. They will utilize approaches such as notifications such as phone calls, texting (SMS), or WhatsApp to take the trial medicine and schedule meetings for stool antigen tests to improve participant engagement.

### Data management {19}

Two assistants will be in charge of data entry and data integrity by double-checking the data. All information will be entered digitally. A Microsoft Access database has data entry forms that will allow the assistants to select specific data from a list of valid data, while what will be stored in the data tables are only the codes that express this data. A main researcher will check the data entry to ensure that the data is entered into the correct fields. Participant files must be stored in a secure location in numerical sequence. After the study is completed, the files will be kept in storage for 3 years. A password system will be used to restrict access to the study data.

### Confidentiality {27}

All data obtained for this trial will be encoded with unique patient identities, ensuring that no individual patient can be recognized. Patient records shall be reviewed only when required and in accordance with Damascus Hospital’s Ethics Committee requirements. Any records relating to participant identification are concealed and will not be made public, to the extent permitted by applicable laws and regulations.

### Plans for collection, laboratory evaluation, and storage of biological specimens for genetic or molecular analysis in this trial/future use {33}

The researchers will collect gastric mucosal biopsy samples, and after 4 weeks + 3 days of end-of-treatment, they will get a stool sample for the HP infection test. For the specimens that bear unique patient identities, a standard methodology will be followed. All biopsies will be sent to the referring hospital’s central pathology laboratory, while stool samples will be sent to the same referring hospital’s central laboratory.

## Statistical methods

### Statistical methods for primary and secondary outcomes {20a}

To compare the eradication rate of HP, we will use the ITT analysis (all individuals who got at least one dosage of the trial treatment) and PPA (all individuals who complied and were tested again using a stool antigen test). We will use the *χ*^2^ test, which will be used for nominal variables, such as sex, treatment protocol, treatment outcome based on stool antigen tests, each adverse event, smoking, and alcoholic status, to determine if there is a relationship between the treatment protocol and the result of the treatment for both the primary and secondary outcomes [50]. We will use the *t*-test to compare the means of the continuous variable, such as patient’s age, with the normal distribution for both the primary and secondary outcomes [51]. The difference in eradication rates between the two treatment regimen groups can be assessed by a two-sided 95% confidence interval (CI). Individuals whose stool antigen tests have not been retested including dropouts will be considered treatment failures. The statistical significance level is a *P*-value of 0.05.

### Interim analyses {21b}

We intend to do a subgroup analysis of the eradication rate of the rescue protocol following first-line therapy failure. We expect rescue treatment to be similarly effective in both groups. The overall eradication rate of the rescue protocol and the relationship between the first-line treatment protocol and the outcome after using the rescue regimen will be assessed by using the *χ*^2^ test, with a two-sided 95% CI of the difference in eradication rates between the two groups. The statistical significance level will be set at a *P*-value of 0.05.

### Methods for additional analyses (e.g., subgroup analyses) {20b}

The overall eradication rate of the rescue protocol and the eradication rate according to the first-line treatment protocol and outcome after using the rescue regimen will be assessed by using the *χ*^2^ test with a two-sided 95% CI of the difference in eradication rates between the two groups. The statistical significance level will be set at a *P*-value of 0.05. Patients who have not had their stool antigen tests reexamined will be deemed treatment failures, i.e., “not eradicated,” in the statistical analysis. Strategies will be put in place to increase follow-up, promote adherence, and prevent missing data. We will report and qualitatively compare the reasons for non-adherence for each randomization group.

### Methods in analysis to handle protocol non-adherence and any statistical methods to handle missing data {20c}

Strategies will be put in place to increase follow-up and promote adherence, like phone calls twice a week, texting, and WhatsApp messaging. To prevent missing data, we will use a programmed Microsoft Access database to enter data. This will allow the use of sound alerts in addition to alert messages that appear when any record is saved with missing data. We will report and qualitatively compare the reasons for non-adherence for each randomization group.

### Plans to give access to the full protocol, participant-level data, and statistical code {31c}

Within a year of publishing the results of this clinical trial, we will upload the entire dataset to a repository to ease sharing, access, and dataset citation.

## Oversight and monitoring

### Composition of the coordinating center and trial steering committee {5d}

The lead investigator is the trial’s designer and is in charge of the study’s execution. The lead investigator and research gastroenterologists are in charge of recruiting, treating, and following up on study participants, as well as reporting severe adverse events and serious, unexpectedly suspected adverse events. The data manager will be in charge of data collection, entry, and verification by matching forms with data within the database. A steering group will be formed to oversee the entire research process. The study team will meet every 2 weeks to monitor the progress of the trial. Regular communication via WhatsApp and phone with patients will ensure the study works successfully.

### Composition of the data monitoring committee, its role, and reporting structure {21a}

Because the trial is short in duration and the treatment regimens are linked to known modest threats, no data monitoring committee will be constituted.

### Adverse event reporting and harms {22}

We will keep track of adverse reactions related to treatment in this trial, which are defined as any events that arise after the administration of the first dosage of the trial drug or any events at baseline that deteriorate in either intensity or frequency after the administration of the first dose of the study drug. An adverse event that meets the threshold for a significant adverse event will be reported to Damascus Hospital’s Ethics Committee according to guidelines [43]. Serious adverse events, which include events that are life-threatening, necessitate inpatient hospitalization, result in persistent or significant disability or incapacity, or may necessitate medical or surgical intervention to prevent one of the outcomes listed above [43]. While the nature and severity of unexpected adverse events do not match the information provided in the appropriate product information [43]. Determination of the relationship between the adverse event and a reasonable chance of being related to treatment exposure. This determination of causality may be based on considerations such as biological plausibility, prior experience with the drug, and the temporal correlation between product exposure and event beginning, as well as dechallenge and rechallenge [43].

### Frequency and plans for auditing trial conduct {23}

An auditor will examine the study protocols for participant enrollment, consent, eligibility, and allocation to research groups; adherence to trial interventions and policies to protect participants, including reporting of harms; and data collection completeness, accuracy, and timeliness. Over the course of the study, the auditor will do at least one onsite monitoring visit every 3 months. The auditing process will be independent of the investigators and the sponsor.

### Plans for communicating important protocol amendments to relevant parties (e.g., trial participants, ethical committees) {25}

Any changes to the protocol that may affect the study’s conduct, such as changes to the potential benefits and risks, will be approved by the Ethics Committee of Damascus Hospital before implementation and communicated to the health authorities in accordance with local regulations.

## Dissemination plans {31a}

The findings of the trial will be made available to clinicians, patients, and the general medical community. The information will be reported irrespective of the magnitude or nature of the treatment's effect. The results will be discussed at national and international conferences, and they will be made available in peer-reviewed journals.

## Discussion

The prevalence of HP infection in Syria is high and is estimated to be about 34% [6]. On the other hand, 67.3% of the Syrian population practiced self-medication, while the most commonly used drugs were antibiotics, which create another problem related to bacterial resistance [52]. Antibiotic resistance restricts the efficacy of triple therapy for HP infections around the world, necessitating searching for new treatment protocols. Until 2018, the most commonly used HP treatment in Syria was triple therapy with clarithromycin and, to a lesser extent, triple therapy with levofloxacin, until a pilot trial was conducted in Syria and proved the ineffectiveness of these regimens [36]. Treatment guidelines recommend the use of levofloxacin-concomitant or bismuth-containing regimens as an alternative first-line treatment, particularly in areas with a high frequency of clarithromycin resistance like Syria [22, 23]. Knowing that levofloxacin concomitant, or levofloxacin sequential, and bismuth-based protocols are the most commonly used for HP infection in Syria. Unfortunately, neither treatment regimen was as effective as expected, since in Syria, the HP eradication rate did not exceed 82% at best [37, 53]. A meta-analysis revealed that a fluoroquinolone-based sequential regimen is a viable therapeutic option for HP infection treatment, with an eradication rate of 87.8%, but it was not evaluated in Syria [40]. As a result, it was critical to find a treatment regimen with a higher eradication rate, and the most important thing was to estimate the eradication rates of a rescue regimen with dual therapy with high-dose PPI and amoxicillin following the failure of the first line [22]. In general, compliance with medication is defined as adherence to taking over eighty percent of prescribed medications. Despite the fact that this regimen is believed to be beneficial, there are concerns about compliance because of the medication change between the first and second weeks [38]. This study will evaluate the two HP treatment regimens that are now of the most interest. Furthermore, because Damascus Hospital is the main hospital linked with the Ministry of Health, we will enroll patients from all throughout Syria. A suitable sample size will be collected to address the trial question statistically. This trial will generate critical evidence that will lead to adjustments in first-line treatment regimens for *Helicobacter pylori* infection in Syria, thus increasing eradication rates and enhancing patient life quality.

## Trial status

The protocol version is V3, December 24, 2023. We did not enroll any patients; recruitment had started in October 2023 and is estimated to end in August 2026. The trial is estimated to end in December 2026.

### Supplementary Information

**Additional file 1.** Sample size calculation.

**Additional file 2.**

## Data Availability

The dataset will be saved on https://data.mendeley.com and will be available within 1 year of the study’s conclusion; we will transfer a totally identified data set to an appropriate data archive for sharing purposes.

## Abbreviations

HP: *Helicobacter pylori*
CI: Confidence interval
ITT: Intention-to-treat
PPA: Per-protocol analyses
PPI/PPIs: Proton-pump inhibitor/inhibitors
NSAIDs: Non-steroidal anti-inflammatory drugs
PPV: Positive predictive value
NPV: Negative predictive value
CI: Confidence interval
SPIRIT: Standard Protocol Items: Recommendations for Interventional Trials
