# Supplementary material for: Efficacy of a 2-week therapy with levofloxacin concomitant versus a levofloxacin sequential regimen for Helicobacter pylori infection in the Syrian population: a study protocol for randomized controlled trial
Source: Trials. 2024 Jan 15;25:55. doi: 10.1186/s13063-024-07906-3 (PMC10789050; doi:10.1186/s13063-024-07906-3)
Supplement: Supplementary file 1 — Additional file 1. Sample size calculation. [file 13063_2024_7906_MOESM1_ESM.docx]

P1= 0.878, the eradication rate of sequential levofloxacin-based treatment regimens based on Meta-analysis [1].

P2= 0.8205, the eradication rate of concomitant levofloxacin-based regimens was based on the results of a randomized clinical trial that was conducted in Syria[2].

We used the following with assumptions (1)80% power (β = 0.2) ;(2) the 5% level of significance (α = 0.05);

The superiority margin is (δ=-0.10) [3].

The difference of ε = 0.20 [3].

Zα = 1.64

Zᵦ = 0.845

Ratio of case to control, K=1

We used the following equation [3,4].

$$N1=KN2={\left( Z\alpha+Z\beta\right)^{2}*\left( P1\left( 1-P1 \right)+P2\left( 1-P2 \right) \right)}/{\left( \varepsilon-\delta\right)^{2}}$$

$$N1=N2={\left( 1.64+0.845 \right)^{2}*\left( 0.877\left( 1-0.877 \right)+0.8205\left( 1-0.8205 \right) \right)}/{\left( 0.2-\left( -0.10 \right) \right)^{2}}$$

N1=N2= 63.32≈64 patients for each treatment group

We also added 15% for possible drop out making the final sample size of 150 patients for two treatment groups.

Reference:

1. Kale-Pradhan PB, Mihaescu A, Wilhelm SM. Fluoroquinolone Sequential Therapy for Helicobacter pylori: A Meta-analysis. Pharmacotherapy: The Journal of Human Pharmacology and Drug Therapy. 2015;35:719–30.

2. Alhalabi M, Alassi MW, Eddin KA, Cheha K. Efficacy of two-week therapy with doxycycline-based quadruple regimen versus levofloxacin concomitant regimen for helicobacter pylori infection: a prospective single-center randomized controlled trial. BMC Infectious Diseases. 2021;21:642.

3. Chung Chow S-, Shao J, Wang H, Lokhnygina Y. Sample Size Calculations in Clinical Research Third Edition. Sample Size Calculations in Clinical Research Third Edition. Third edition. © 2018 by Taylor & Francis Group, LLC CRC Press is an imprint of Taylor & Francis Group, an Informa business; 2018. p. 71–81.

4. Wang X, Ji X. Sample Size Estimation in Clinical Research: From Randomized Controlled Trials to Observational Studies. Chest. 2020;158:S12–20.
